# Effects of *Escherichia coli* and *Proteus mirabilis* on the Growth and Aggregation of Calcium Oxalate Crystal under Microaerobic Conditions

**DOI:** 10.3390/diagnostics12112651

**Published:** 2022-10-31

**Authors:** Krittaya Saelee, Aroonlug Lulitanond, Nattaya Sae-ung, Vitoon Prasongwatana, Patcharee Boonsiri, Ratree Tavichakorntrakool

**Affiliations:** 1 Centre for Research and Development of Medical Diagnostic Laboratories, Faculty of Associated Medical Sciences, Khon Kaen University, Khon Kaen 40002, Thailand; 2 School of Medical Technology, Faculty of Associated Medical Sciences, Khon Kaen University, Khon Kaen 40002, Thailand; 3 Department of Biochemistry, Faculty of Medicine, Khon Kaen University, Khon Kaen 40002, Thailand

**Keywords:** *Escherichia coli*, *Proteus mirabilis*, co-culture, kidney stones, calcium oxalate monohydrate, crystal growth, crystal aggregation

## Abstract

*Escherichia coli* and *Proteus mirabilis* are common single- and polymicrobial urinary tract infections which can survive under various oxygen levels, including inside of stone matrices. Therefore, we aimed to investigate and compare the calcium oxalate monohydrate (COM) lithogenic activities including COM crystal growth and aggregation under microaerobic conditions of *E. coli* and *P. mirabilis* isolated from the same stone matrix. The crystal growth was analyzed as the delta crystal area while the crystal aggregation was analyzed as the number of crystal aggregates. The results showed that compared to blank control, *E. coli*, *P. mirabilis* and the co-culture of *E. coli* and *P. mirabilis* were able to significantly promote COM crystal growth under microaerobic conditions. Interestingly, the delta crystal area in the co-culture under microaerobic conditions was larger than that of *E. coli* alone and *P. mirabilis* alone. In addition, only *P. mirabilis* alone and the co-culture were able to significantly increase COM aggregates. This study demonstrated that single- and co-culture of *E. coli* and *P. mirabilis* could promote COM crystal growth and aggregation under microaerobic conditions. The co-culture of *E. coli* and *P. mirabilis* may provide the combination effect on COM crystal interactions. The bacterial surfaces and the important effects on bacteria–crystal interactions should be further evaluated.

## 1. Introduction

Kidney stone disease, especially the calcium stone type, is a common public health problem worldwide, including in the Northeastern region of Thailand [1,2,3,4]. Calcium oxalate (CaOx) stone formation mainly starts with crystal nucleation, growth, aggregation, and retention. CaOx is a calcium salt of the oxalate group which can occur in human urine. However, the imbalance of crystal promoters and inhibitors were crucial for kidney stone development [5,6,7]. Calcium oxalate monohydrate (COM; CaC_2_O_4_·H_2_O), the least soluble form of CaOx, is found more commonly in stones than calcium oxalate dihydrate (COD; CaC_2_O_4_·2H_2_O) [7,8]. Several factors involved with kidney stone disease were also evaluated in this region such as K and Mg depletion in skeletal muscles [9,10] and hypocitraturia [11]. Moreover, hematuria can be found in kidney stone disease and urinary tract infection, leading to the evaluation of red blood cell effects on the stone genesis. The results showed that red blood cell membrane fragments, but not intact red blood cells, showed the promoting effect on calcium oxalate monohydrate (COM) crystal growth and aggregation [12,13]. Urinary tract infection (UTI) is a well-known risk factor for magnesium ammonium phosphate (struvite) stones or infection-induced stones [14]. *Proteus mirabilis* and other urease-producing bacteria can catalyze the hydrolysis of urea and increase the pH of urine, leading to a suitable environment to form struvite crystals, a non-calcium crystal type [14,15]. However, our previous study [16] showed that 45 bacteria were isolated from urine and/or stones of 36 kidney stone patients. Most of the bacteria were isolated from metabolic stones, especially CaOx. The top two most common bacteria isolated from the stone matrices were *Escherichia coli* (a non-citrate-utilizing and non-urea-splitting bacterium) and *P. mirabilis* (a citrate-utilizing and urea-splitting bacterium), respectively. Interestingly, some subjects had positive polybacterial cultures from their stone matrices. Both *E. coli* and *P. mirabilis*, categorized in the same order as Enterobacterales [17], are Gram-negative and facultative anaerobic bacteria. They can grow and adapt themselves in various oxygen levels [18,19,20,21]. Many studies have revealed that bacteria experience a limited amount of oxygen during urinary tract infection [22,23,24]. In healthy subjects, the mean bladder urine pO_2_ ranged from 23–45 mmHg [25]. Moreover, there is less oxygen concentration around 30 and 10 mmHg in the renal cortex and medulla, respectively [26]. Therefore, it is likely that bacteria have limited oxygen for their metabolism, which may be involved in crystal processes during UTI and on their way to the urological system. Our previous study showed that the cellular proteome findings revealed the differential levels of proteins involved in stress response, RNA/protein, and carbohydrate metabolisms in *E. coli* isolates from stone matrices that might be an adaptive response of *E. coli* from remote infection to survive within the stone matrices [27]. The information led us to question the roles of these bacteria isolated from stone matrices in stone formation under microaerobic condition. In addition, there is limited information about the effects of these bacterial single- and co-infections on CaOx crystal growth and aggregation under microaerobic conditions, which may better represent the urological system or the inside of the stone matrices. 

Therefore, this study aimed to investigate COM lithogenic activities under microaerobic, single- and co-culture conditions of *E. coli* and *P. mirabilis* isolated from the same stone matrix.

## 2. Materials and Methods

### 2.1. Sample Collection and Bacterial Properties 

This study was conducted following the Declaration of Helsinki and approved by the Institutional Ethical Committee of Khon Kaen University, Khon Kaen, Thailand (Approval Nos. HE581501 and HE 621366).

Stone samples were collected from a total of 100 kidney stone formers in the Northeastern region of Thailand [16] who were admitted to the hospital for elective kidney stone removal by percutaneous nephrolithotomy. These 36 subjects had bacterial isolates cultivated from their catheterized urine and/or stone matrices [16]. *E*. *coli* and *P*. *mirabilis* were the top two most common bacteria isolated from the stone matrices. Moreover, polybacterial isolates were also found in some stone culture samples [16]. Therefore, *E*. *coli* and *P*. *mirabilis* isolates from the same stone matrix (Table 1) were used to investigate the COM crystal growth and aggregation.

### 2.2. Negative and Positive Controls

From the previous studies [12,13], the intact red blood cells had no promoting effects on COM crystal growth and aggregation while red blood cell membrane fragments showed the promoting effect on COM crystal growth and aggregation. Therefore, intact red blood cells and red blood cell membrane fragments were used as negative and positive controls, respectively, for COM crystal growth and aggregation. 

### 2.3. Bacterial Culture Conditions

*E. coli* and *P. mirabilis* isolated from stone matrices were used to evaluate the CaOx lithogenic activities [16]. *E. coli* and *P. mirabilis* were cultured on MacConkey agar at 37 °C overnight and sub-cultured in Luria–Bertani (LB) broth at 37 °C for 24 h.

### 2.4. Setting of Microaerobic Conditions

Microaerobic conditions with 6–12% of O_2_ and 5–8% of CO_2_ were generated in an anaerobic rectangular jar (Thermo Scientific, Tokyo, Japan) using AnaeroPack-MicroAero (Mitsubishi Gas Chemical Company, Tokyo, Japan). *Helicobacter pylori* (a microaerophilic bacterium) was used as an indicator for microaerobic condition.

### 2.5. Preparation of Calcium Oxalate Monohydrate (COM) Crystal

COM seeds were prepared by mixing calcium chloride dihydrate and sodium oxalate at final concentrations of 5.0 and 0.5 mM [28], in a buffer containing 10 mM tris hydrochloride (pH 7.4) and 90 mM sodium chloride. The solution was left at 25–30 °C overnight and then centrifuged at 1500 rpm for 10 min [28,29]. The crystals were washed three times with methanol. After the last washing, the supernatant was discarded, and the crystal pellets were dried at 25–30 °C overnight.

### 2.6. Evaluations of Bacteria on COM Crystal Growth

To investigate the COM growth with a slightly modified method [28], the final concentration of 5.0 mM calcium chloride dihydrate and 0.5 mM sodium oxalate in buffer containing 10 mM Tris hydrochloride and 90 mM sodium chloride (pH 7.4) was prepared in a six-well plate. The solutions were then incubated at 37 °C under microaerobic conditions. After one-hour incubation (T_0_), a final concentration of 10^5^ cells/mL intact red blood cells (negative control), 10^5^ cells/mL red blood cell membrane fragments (positive control) [12,13] or 10^5^ CFU/mL bacteria were added into each well. For the co-culture, each *E*. *coli* and *P*. *mirabilis* (10^5^ CFU/mL, a final concentration of each) was added at a ratio 1:1. The solution with nothing added served as the blank control. Then, the plates were incubated again at 37 °C for one hour (T_1_) under microaerobic conditions. At T_0_ and T_1_, the 15 low power fields (LPF) of crystal images were randomly taken for 100 crystals/LPF/test. COM crystal sizes at T_0_ and T_1_ were then measured using ImageJ software (Version 1.52a, US National Institutes of Health, Bethesda, MD, USA). The crystal growths (represented by Δ Crystal area; µm^2^) were calculated following this formula:

Δ Crystal area (µm^2^) = Crystal area at T_1_ − Crystal area at T_0_

### 2.7. Preparation of Saturated Aggregation Buffer (SAB)

The artificial urine was prepared following components: 12.136 g of urea, 0.172 g of uric acid, 0.452 g of creatinine, 1.484 g of trisodium citrate dihydrate, 3.172 g of sodium chloride, 2.252 g of potassium chloride, 0.804 g of ammonium chloride, 0.444 g of calcium chloride dihydrate, 0.500 g of magnesium sulfate heptahydrate, 0.172 g of sodium bicarbonate, 0.016 g of sodium oxalate, 1.292 g of sodium sulfate, 0.564 g of sodium dihydrogen phosphate dihydrate, and 0.140 g of disodium hydrogen phosphate dissolved in 1 L of deionized water. The artificial urine pH was adjusted to 6.0 and then saturated with calcium and oxalate ions by adding COM crystals into the artificial urine until the crystals could not dissolve further [30]. After that, the solution was filtered with 0.2 µm sterile polyether sulfone filter paper (Whatman Ltd., United Kingdom) and collected to use for COM crystal aggregation assay.

### 2.8. Evaluations of Bacteria on COM Aggregation

In a slightly modified method for crystal aggregation [30], the final concentrations of COM crystals (100 µg/mL) were added into all wells of six-well plate containing SAB. Then, the final concentrations of intact red blood cells (10^5^ cells/mL), red blood cell membrane fragments (10^5^ cells/mL) or bacteria (10^5^ CFU/mL) were added as negative, positive controls or under single-culture conditions, respectively. For the co-culture, each *E*. *coli* and *P*. *mirabilis* (10^5^ CFU/mL, a final concentration of each) was added at a ratio 1:1. The solution with nothing added served as the blank control. The solutions were incubated at 37 °C for one hour under microaerobic conditions, and then evaluated under an inverted light microscope (CK2, Olympus, Tokyo, Japan). The 45 LPF/well were examined and pictures were captured. Crystal aggregates were defined as two or more crystals that adhered together. 

### 2.9. Statistical Analysis

All experiments were performed in triplicate (three independent experiments). Quantitative data were shown as the mean and standard deviation (mean ± SD). Multiple comparisons were analyzed using variance with Bonferroni. A *p*-value of less than 0.05 was statistically significant. Statistics were calculated by SPSS program (IBM Corp, Armonk, NY, USA).

## 3. Results

### 3.1. Involvement of Bacteria on Calcium Oxalate Monohydrate (COM) Crystal Growth under Microaerobic Condition

**Figure 1:**
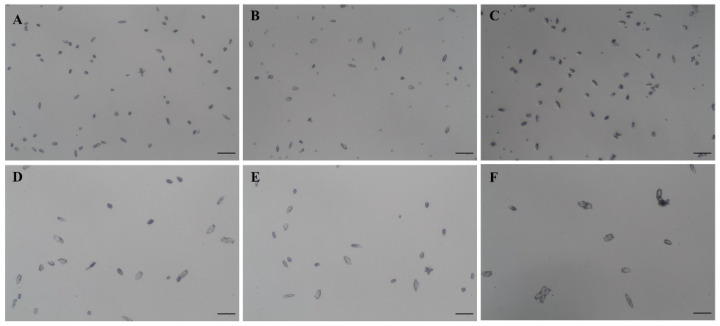
COM crystal growth morphology under microaerobic of the blank control (**A**), negative control (**B**), positive control (**C**), *E. coli* (**D**), *P. mirabilis* (**E**), and co-cultured *E. coli* and *P. mirabilis* (**F**). Original magnification and scale bar (―) were 100× and 50 µm, respectively.

The crystal growth was analyzed as the delta crystal area (crystal area at T_1_ – crystal area at T_0_, µm^2^) and the ratio of the mean of delta crystal area. Intact red blood cells (RBC) and RBC membrane fragments were used as negative and positive controls, respectively. *E. coli* alone, *P. mirabilis* alone, and the co-culture of *E. coli* and *P. mirabilis* were analyzed. The condition without bacteria, intact RBC and RBC membrane fragments served as a blank control. The negative control (3.90 ± 6.77 µm^2^) had no effect on crystal growth (comparable to the blank control (3.83 ± 5.90 µm^2^)), while the positive control (95.52 ± 46.55 µm^2^) clearly enhanced crystal growth. This is the first dataset to show that the delta crystal areas of *E. coli*, *P. mirabilis* and co-culture of *E. coli* and *P. mirabilis* under microaerobic condition were significantly larger than the blank control (*p*-value < 0.001, < 0.001. < 0.001, respectively) (Table 2, Figure 1). Interestingly, the delta crystal area of a co-culture of *E. coli* and *P. mirabilis* was significantly larger than that of *E. coli* alone and *P. mirabilis* alone (*p*-value < 0.001 and < 0.001, respectively). Indeed, co-culture of *E. coli* and *P. mirabilis* resulted in the most potential activity of crystal growth, which was represented by the ratio of the mean of delta COM crystal area between samples and the blank control.

### 3.2. Involvement of Bacteria on COM Crystal Aggregation under Microaerobic Conditions

**Figure 2:**
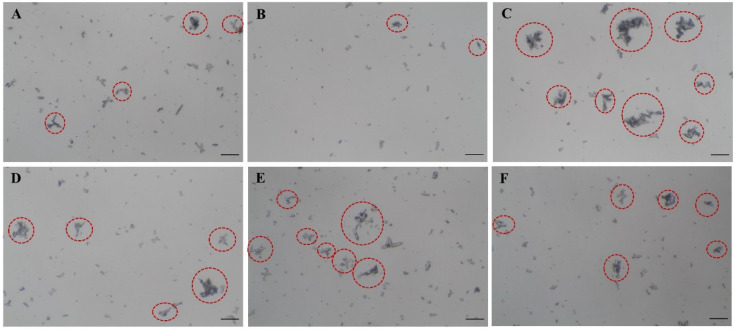
COM crystal aggregation morphology under microaerobic of the blank control (**A**), negative control (**B**), positive control (**C**), *E. coli* (**D**), *P. mirabilis* (**E**), and co-cultured *E. coli* and *P. mirabilis* (**F**). Original magnification and scale bar (―) were 100× and 50 µm, respectively.

The crystal aggregation was analyzed as the number of crystal aggregates. After one-hour incubation under microaerobic conditions, COM crystal aggregation was evaluated. As estimated, the negative control (1.98 ± 1.57/LPF) had no effect on crystal aggregates (comparable to the blank control (2.18 ± 1.54/LPF)), while the positive control (4.53 ± 3.96/LPF) clearly enhanced COM crystal aggregates. The numbers of COM crystal aggregates of *P*. *mirabilis* and a co-culture of *E. coli* and *P. mirabilis* under microaerobic conditions were significantly increased compared to the blank control (*p*-value < 0.05 and < 0.001, respectively) (Table 3, Figure 2). Remarkably, *P. mirabilis* alone and the co-culture of *E*. *coli* and *P*. *mirabilis* had tended to enhance the potential activity of crystal aggregation, which was represented by the ratio of mean of COM aggregates between bacteria and the blank control.

## 4. Discussion

From our previous data [16], *E. coli* and *P. mirabilis* were the top two most common bacteria isolated from the stone matrices. Indeed, *E. coli* (a non-citrate-utilizing, non-urea-splitting, and motility bacterium) and *P. mirabilis* (a citrate-utilizing, urea-splitting, and swarming motility bacterium) isolated from the same stone matrix were used to study the crystal growth and aggregation under microaerobic culture for mimicking the urinary system. They can grow at a low oxygen level [18,19,20,21]. Moreover, their antimicrobial susceptibility patterns were susceptible to amikacin, trimethoprim/sulfamethoxazole, ceftazidime, cephalothin, gentamicin, and norfloxacin, which were consistent with the previous report about common *E*. *coli* and *P*. *mirabilis* uropathogens in Lebanon [31].

The results indicated that the delta crystal areas (crystal growth) of *E. coli* alone under microaerobic conditions were significantly larger than the blank and negative controls, while the number of COM crystal aggregates (crystal aggregation) was increased, but there was no statistically significant difference. The data were rather similar to a previous report that demonstrated that intact viable *E. coli* could promote CaOx crystal growth and aggregation under aerobic conditions [32]. The difference of bacterial strains, methods, and oxygen level may have influences on the levels of COM crystal aggregates. Our previous study by Amimanan et al., 2017 [33] showed that a molecular mechanism of *E. coli* promoted CaOx crystal growth and aggregation may be involved with an elongation factor Tu on the surface of the outer membrane vesicles’ interaction with calcium ions. This *E. coli* strain could motile; therefore, the flagella may be a factor that promoted the CaOx crystal growth and aggregation [34]. Moreover, an in vivo experiment showed that *E. coli* plays a significant role in CaOx stone pathogenesis. COM crystals could be aggregated and adhered by *E*. *coli* at medulla of kidney tissue with limiting oxygen concentration. Possible mechanisms were speculated, including bacteria or bacterial biofilms involved in stone formation [35]. 

In the same way, our results also showed that the delta crystal areas and COM crystal aggregates of *P. mirabilis* alone under microaerobic conditions were significantly larger than the blank and negative controls. In a previous study using *Klebsiella pneumoniae*, urea-splitting and a capsule bacterium were shown to promote CaOx crystal growth and aggregation under aerobic conditions [32]. This *P. mirabilis* strain is a Gram-negative facultative anaerobe with swarming motility and strong urease producing [36]. Kanlaya et al., 2019 [34] showed that bacterial components (e.g., flagella and capsule) could promote the CaOx crystal growth and aggregation.

Interestingly, the delta crystal areas and numbers of CaOx crystal aggregates of the co-culture of *E. coli* and *P. mirabilis* under microaerobic conditions were significantly larger than the blank and negative controls. Moreover, the delta crystal areas of the co-culture of *E. coli* and *P. mirabilis* were also significantly larger than those of *E. coli* alone and *P. mirabilis* alone. However, the roles of co-culture of *E. coli* and *P. mirabilis* in CaOx stone genesis need to be evaluated. It is challenging for researchers to illustrate the epidemiology of polymicrobial urine cultures [37]. The co-culture of *E*. *coli* and *P*. *mirabilis* epidemiology in urine and stone cultures should be further investigated. Both of the *E. coli* and *P. mirabilis* strains used in this study could motile via flagella. The bacterial surface (e.g., OMV [33] and flagella [34]) may be the important effects on bacteria–crystal interactions. The previous study showed that the cell number of the *P. mirabilis* alone was lower than the co-culture of *P. mirabilis* and *E. coli* [36,37,38]. This study indicated that *P. mirabilis* could grow and colonize better in the presence of *E. coli*. This co-culture of *E. coli* and *P. mirabilis* should be considered for the persistence of bacterial growth in the urinary tract which may have a combined effect on CaOx crystal growth and aggregation. If they retained inside the stone matrix, they may act as a reservoir to cause recurrent infection and enhance the oxidative stress and inflammation in renal cells. A recent study by An et al., 2021 demonstrated that *E. coli* could promote CaOx stone genesis by enhancing oxidative injury and inflammation regulated by the PPK1/flagellin, which stimulated the pathways of NF-κB/P38 [29]. Moreover, type 1 fimbriae and MR/P fimbriae of *E. coli* and *P. mirabilis*, respectively, may increase the expression during oxygen limitation. It may be involved in the disease progression within the oxygen-restriction in urinary tract system [20]. 

This in vitro study provides a preliminary explanation on the mechanism of CaOx lithogenic activities of single- and co-culture of *E. coli* and *P. mirabilis*. Our information provides some new points for further investigation, especially bacterial surface and flagella, the important effects on bacteria–crystal interactions.

However, there are some limitations in our present study. Firstly, all the experiments were done entirely in vitro (not in vivo condition) which means that the microenvironments are different from those in the urinary system. Secondly, the in vitro experiment had no urine flow and stone modulators are the important factors that can affect crystal processes and UTI. Lastly, we measured the delta crystal area which is an updated parameter that is acceptable as the crystal size in crystal growth [28,33,34,39]. However, the three-dimensional crystal analysis should be further investigated.

## 5. Conclusions

Our important findings provide an explanation on the mechanism of COM lithogenic activities of *E. coli* and *P. mirabilis*. Intact single and co-culture of *E. coli* and *P. mirabilis* could promote COM crystal growth and aggregation under microaerobic conditions. The co-culture of *E. coli* and *P. mirabilis* should be considered as the combination effect on COM crystal interactions. The important factors on bacteria–crystal interactions, including the bacterial surface and flagella should be further evaluated.

## Figures and Tables

**Table 1:** The properties of *E. coli* and *P. mirabilis* isolates from the same stone matrix.

Clinical Bacterial Strains	C	U	M	Antimicrobial Susceptibility								
				AK	AMP	CF	SXT	GM	NOR	OFX	CTX	CAZ
*E*. *coli*	−	−	+	S	S	S	S	S	S	S	S	S
*P*. *mirabilis*	+	+	+	S	S	S	S	S	S	S	S	S

C = Citrate-utilizing, U = Urea-splitting, M = Motility; − = Negative, + = Positive; S = Susceptible, R = Resistant, AK = Amikacin; AMP = Ampicillin; CF = Cephalothin; SXT = Sulfamethoxazole/trimethoprim; GM = Gentamicin; NOR = Norfloxacin; OFX = Ofloxacin; CTX = Cefotaxime; CAZ = Ceftazidime.

**Table 2:** Effect of bacteria on COM crystal growth under microaerobic conditions.

Parameters	Mean of Delta COM Crystal Area (µm^2^; mean ± SD)	Ratio of Mean of Delta COM Crystal Area Between Samples and Blank Control
Blank control	3.83 ± 5.90	1.00
Negative control	3.90 ± 6.77	1.02
Positive control	95.52 ± 46.55 *^,^ **^,^^†^	24.94
*E* *. coli*	88.69 ± 21.17 *^,^ **^,^^†^	23.16
*P* *. mirabilis*	85.30 ± 41.72 *^,^ **^,^^†^	22.27
Co-culture of *E. coli* and *P. mirabilis*	194.81 ± 105.00 *^,^ **	50.86

*, ** and ^†^ = Statistical significance (*p*-value < 0.05 when compared with blank, negative controls, and co-culture of *E. coli* and *P. mirabilis*, respectively).

**Table 3:** Effect of bacteria on COM aggregates under microaerobic conditions.

Parameters	Number of COM Aggregates/LPF (mean ± SD)	Ratio of Mean of COM Aggregates between Samples and Blank Control
Blank control	2.18 ± 1.54	1.00
Negative control	1.98 ± 1.57	0.91
Positive control	4.53 ± 3.96 *^,^ **	2.08
*E* *. coli*	2.98 ± 1.70	1.37
*P . mirabilis*	3.80 ± 1.89 *^,^ **	1.74
Co-culture of *E. coli* and *P. mirabilis*	4.11 ± 1.56 *^,^ **	1.88

* and ** = Statistical significance (*p*-value < 0.05 when compared with blank and negative controls, respectively).

## Data Availability

All data are available in this publication.
